# Early changes of bone metabolites and lymphocyte subsets may participate in osteoporosis onset: a preliminary study of a postmenopausal osteoporosis mouse model

**DOI:** 10.3389/fendo.2024.1323647

**Published:** 2024-02-28

**Authors:** Sizhu Wang, Yuyou Qiu, Cuisong Tang, Huan Tang, Jinchuan Liu, Jieying Chen, Lin Zhang, Guangyu Tang

**Affiliations:** ^1^ Department of Radiology, Shanghai Tenth People’s Hospital, Tongji University School of Medicine, Shanghai, China; ^2^ Department of Radiology, Guangdong Provincial People’s Hospital (Guangdong Academy of Medical Sciences), Southern Medical University, Guangzhou, Guangdong, China; ^3^ Department of Radiology, Clinical Medical College of Shanghai Tenth People’s Hospital of Nanjing Medical University, Shanghai, China; ^4^ Department of Radiology, Huadong Hospital of Fudan University, Shanghai, China; ^5^ Department of Obstetrics and Gynaecology, Li Ka Shing (LKS) Faculty of Medicine, The University of Hong Kong, Hong Kong, Hong Kong SAR, China

**Keywords:** osteoporosis, metabolism, LC-MS/MS, lymphocyte, B cell

## Abstract

**Purpose:**

Metabolic and immune changes in the early stages of osteoporosis are not well understood. This study aimed to explore the changes in bone metabolites and bone marrow lymphocyte subsets and their relationship during the osteoporosis onset.

**Methods:**

We established OVX and Sham mouse models. After 5, 15, and 40 days, five mice in each group were sacrificed. Humeri were analyzed by microCT. The bone marrow cells of the left femur and tibia were collected for flow cytometry analysis. The right femur and tibia were analyzed by LC-MS/MS for metabolomics analysis.

**Results:**

Bone microarchitecture was significantly deteriorated 15 days after OVX surgery. Analysis of bone metabolomics showed that obvious metabolite changes had happened since 5 days after surgery. Lipid metabolism was significant at the early stage of the osteoporosis. The proportion of immature B cells was increased, whereas the proportion of mature B cells was decreased in the OVX group. Metabolites were significantly correlated with the proportion of lymphocyte subsets at the early stage of the osteoporosis.

**Conclusion:**

Lipid metabolism was significant at the early stage of the osteoporosis. Bone metabolites may influence bone formation by interfering with bone marrow lymphocyte subsets.

## 1 Introduction

Osteoporosis is one of the most common bone diseases, which has become a major public health problem (1). Osteoporosis causes enormous health and financial consequences due to osteoporotic fractures (2). Age and gender are both important predictors of osteoporosis. Postmenopausal women are more susceptible to osteoporosis. Estrogen withdrawal contributes to the loss of bone and bone microarchitecture deterioration (3). Metabolite alterations have been found to happen in the fecal and plasma in osteoporosis, which help provide information for the diagnosis, mechanism, therapy, and prevention of osteoporosis (4–6).

Metabolites are significant for essential functions, including energy production, signal transduction, and apoptosis, which enables a wide range of functions in cells and organisms (7). Metabolism has become a new research hotspot. Untargeted and targeted spectrometry are two main kinds of methods to identify metabolites. Compared with targeted metabolomics, untargeted metabolomics could measure the broadest range of metabolites without *a priori* knowledge of the metabolites, which is more suitable for preliminary exploratory research (8). Untargeted metabolomics have been applied in various disease studies, such as diabetes, non-alcoholic fatty liver disease, and pediatric chronic kidney disease (9–11). Metabolic pathway disorders were also discovered in osteoporosis, and metabolic alterations were related with the efficacy of antiosteoporosis drugs (12–14).

Immune status is related with the loss of bone mass and deterioration of bone microstructure (15). Osteoimmunology has also become a research hotspot since the skeletal and immune systems share a lot of cytokines and signaling molecules (16). Lymphocytes are important regulators of bone turnover homeostasis (17). Immune cells could interact with osteoblasts and osteoclasts mostly by paracrine (18). Many T-lymphocyte subsets, such as Th cells, natural killer T cells, and CD8 T cells, are involved in the bone homeostasis (19). Interferon (IFN)-γ, mainly produced by Th1 cells, is one of the inhibitors of bone loss. TNF-α could contribute to the osteoclasts formation by acting on osteoclasts and RANKL (20). Th17 cells, which could produce IL-17, RANKL, and TNF, were observed to be related with sex steroid deficiency-associated bone loss. The migration of Th17 cells from gut to the bone marrow contributed to the postmenopausal osteoporosis (21, 22). B cells are also a significant regulator of osteoclast formation by the RANK/RANKL/OPG axis, granulocyte colony-stimulating factor, β-catenin, and so on (18, 23, 24). In addition, it suggested that estrogen could modulate the immune-cell regulation of bone loss. Bone loss induced by estrogen deficiency was partly driven by adaptive immune function (25, 26).

However, there are few studies that investigate the changes in bone metabolites as well as bone marrow lymphocyte subsets during the onset of the osteoporosis. In this study, we applied untargeted metabolomics analysis to detect the changes of bone metabolites and flow cytometry to analyze the changes of bone marrow lymphocyte subsets at different time points after the OVX surgery or Sham surgery. Correlation between these parameters was also explored, providing a new insight into the mechanism of osteoporosis onset.

## 2 Methods

### 2.1 Animals and osteoporotic model method

The Animal Ethics Committee of Shanghai Tenth People’s Hospital ratify the experimental protocol and animals feeding method in this study. A total of 30 female C57BL/6 mice (10 weeks old) were purchased from Vital River Laboratory Animal Technology Co., Ltd. Mice were fed freely and accommodated in a temperature- and humidity-controlled room environment with a 12/12-h light/dark cycle. Mice were indiscriminately classified into ovariectomized (OVX) and Sham operated groups. Isoflurane was used for anesthesia. Bilateral ovariectomy was performed in the OVX group. After the mice were anesthetized with isoflurane, the abdomen was opened and the abdominal muscles were gently separated. The oviduct was ligated, and the ovary was removed. The same process was performed on the other side. A sham surgery was performed in the Sham group.

### 2.2 Specimen collection

On the 5th, 15th, and 40th days after surgery, five mice in the OVX group and Sham group were sacrificed respectively. The left humerus and bilateral femur and tibia were separated. The muscles and soft tissues were removed. Humeri were fixed in 4% paraformaldehyde solution for 48 h and then put in 75% alcohol at 4°C for reservation and subsequent microCT scan. To isolate the bone marrow cells, the left femur and tibia were rinsed with 1 mL of RPMI 1640 medium (5% FBS) using a sterile syringe. The cell suspension was passed through a 70-μm filter and centrifuged at 500g for 5 min. To lyse the red blood cells, 1 mL of red blood cell lysate per mouse was added on ice for 3 min and then the reaction was stopped by adding 10 mL of PBS. The cells were washed with PBS once and resuspended in PBS after centrifugation at 500g for 5 min for flow cytometry analysis. The right femur and tibia were stored in a −80°C refrigerator for metabolomics analysis.

### 2.3 Bone microarchitecture measurement

The fixed and preserved bone samples were scanned by the SkyScan 1176 small animal micro-CT scanning image system (Bruker, Germany), with a resolution of 18 μm. The microstructure parameters of bone tissue in the target area were analyzed by systematic analysis software, and the analysis area of all samples was consistent.

### 2.4 Metabolomics profiling

A bone sample was ground with a grinding bead. A 400-μL extraction solution (methanol: water = 4:1 (v:v)) and an internal standard (L-2-chlorophenylalanine) were applied for metabolite extraction. Samples were ground by the Wonbio-96c frozen tissue grinder for 6 min (−10°C, 50 Hz), followed by low-temperature ultrasonic extraction for 30 min (5°C, 40 kHz). The bone samples were stored at −20°C for 30 min and then centrifuged for 15 min (4°C, 13,000 g). The supernatant was collected for LC-MS/MS analysis by a Thermo UHPLC Q Exactive HF-X system equipped with an ACQUITY HSS T3 column (100 mm × 2.1 mm i.d., 1.8 μm; Waters, USA). The mass spectrometric data were collected by a Thermo UHPLC Q Exactive HF-X Mass Spectrometer equipped with an electrospray ionization (ESI) source operating in positive mode and negative mode. The optimal conditions were set as follows: source temperature at 425°C; sheath gas flow rate at 50 arb; Aux gas flow rate at 13 arb; ion-spray voltage floating (ISVF) at −3,500 V in negative mode and 3,500 V in positive mode, respectively; normalized collision energy, 20–40–60 V rolling for MS/MS. Full MS resolution was 60,000, and MS/MS resolution was 7,500. Data acquisition was performed with the data-dependent acquisition (DDA) mode. The detection was carried out over a mass range of 70–1,050 m/z.

### 2.5 Data processing and multivariate statistical analysis

The metabolic profile data matrix was input into the online platform of Majorbio Cloud Platform (www.majorbio.com) for data preprocessing and multivariate statistical analysis (27). Features with missing values greater than 50% in each group were deleted. Other missing values are filled in using the mean value. The response intensities of the sample mass spectrometry peaks were normalized using the sum normalization method. The variables of QC samples with relative standard deviation (RSD) >25% were excluded. A multivariate analysis (OPLS-DA) was applied to present the sample distribution in the OVX group and Sham group by R package “ropls” (version 1.6.2). Metabolites with variable importance projection (VIP) value ≥1and a p value <0.05 were regarded as significant metabolites. The identified metabolites were mapped into their biochemical pathways through metabolic enrichment and pathway analysis based on the KEGG database (http://www.genome.jp/kegg/). Enrichment analysis was used to obtain the most relevant biological pathways for different groups by Python packages “scipy. stats” (https://docs.scipy.org/doc/scipy/).

### 2.6 Flow cytometry analysis of immune cells in bone marrow

The cell suspension was initially treated with CD16/32 (BioLegend, USA, 101302) to block Fc receptors at 4°C for 15 min. After one wash with PBS, the cells were labeled with Zombie Aqua (BioLegend, USA, 423101) and fluorochrome-conjugated monoclonal antibodies provided in **Supplementary Table S1** at 4°C for 30 min. Following another wash with FACS buffer, the cells were resuspended in FACS buffer and analyzed using a FACS analyzer (Beckman Coulter CytoFLEX™).

### 2.7 Statistical analysis

Shapiro–Wilk test was used to assess whether data were normally distributed. Data were expressed as mean ± standard error of the mean (SEM) as data were normally distributed continuous variables. Student’s unpaired t-test was used to evaluate the differences between two groups, and Spearman rank correlation analysis was applied to evaluate the relationship between two parameters by GraphPad prism 8 software. A value of *p < 0.05, **p < 0.01, and ***p < 0.001 was considered to indicate a statistically significant difference.

## 3 Results

### 3.1 Bone microarchitecture parameter analysis

**Figure 1:**
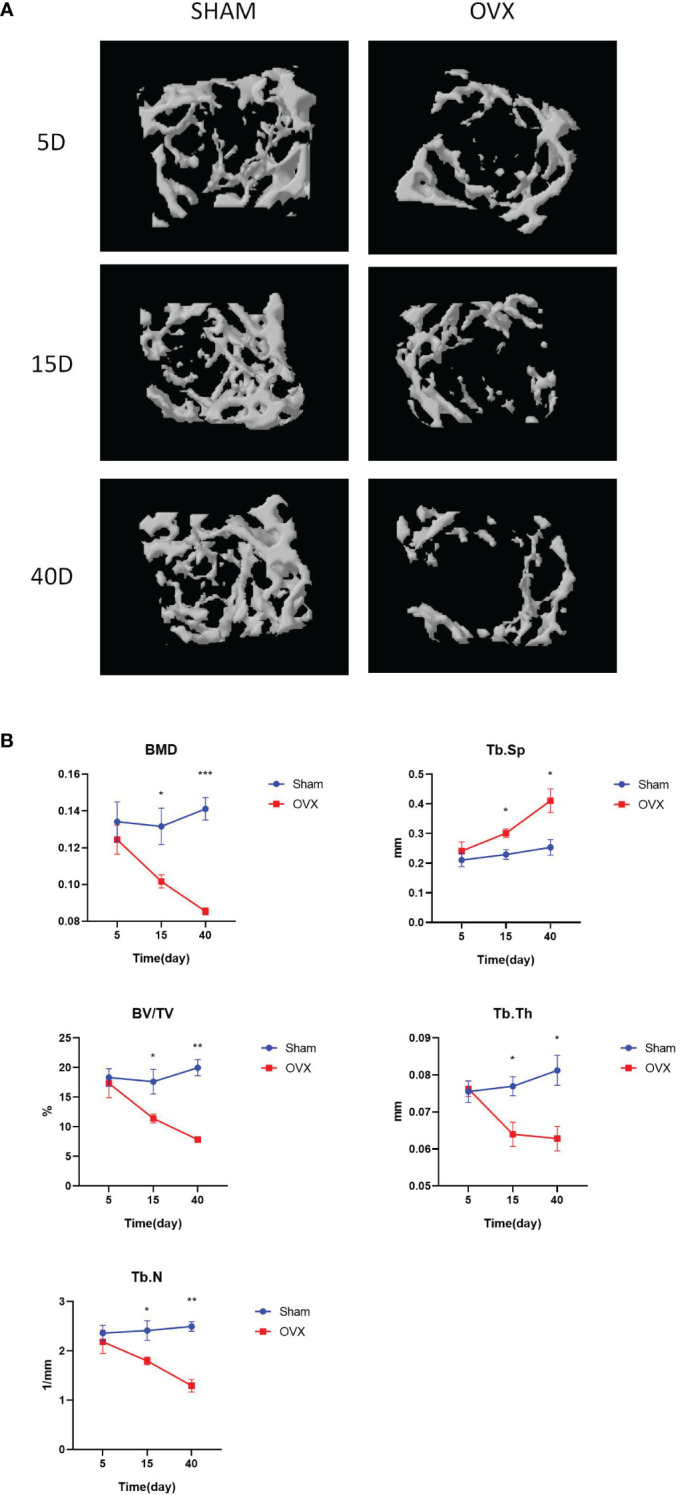
Construction of the osteoporosis mouse model. **(A)**. The typical microCT images of humerus. **(B)**. The microarchitecture parameters of humerus of mice after OVX or Sham surgery at different time points. *p < 0.05, **p < 0.01, and ***p < 0.001.

Typical microCT images of the humerus at different time points after surgery are shown in **Figure 1A**. Compared with the Sham group, the humeral trabeculae in the OVX group were sparse over time. The values of BMD, BV/TV, Tb.Th, and Tb.N were significantly decreased in the OVX group since 15 days after surgery compared with the Sham group, whereas the value of Tb.Sp was significantly increased in the OVX group since 15 days after surgery (**Figure 1B**). The difference became more obvious 40 days after surgery.

### 3.2 Metabolic profiling analysis

**Figure 2:**
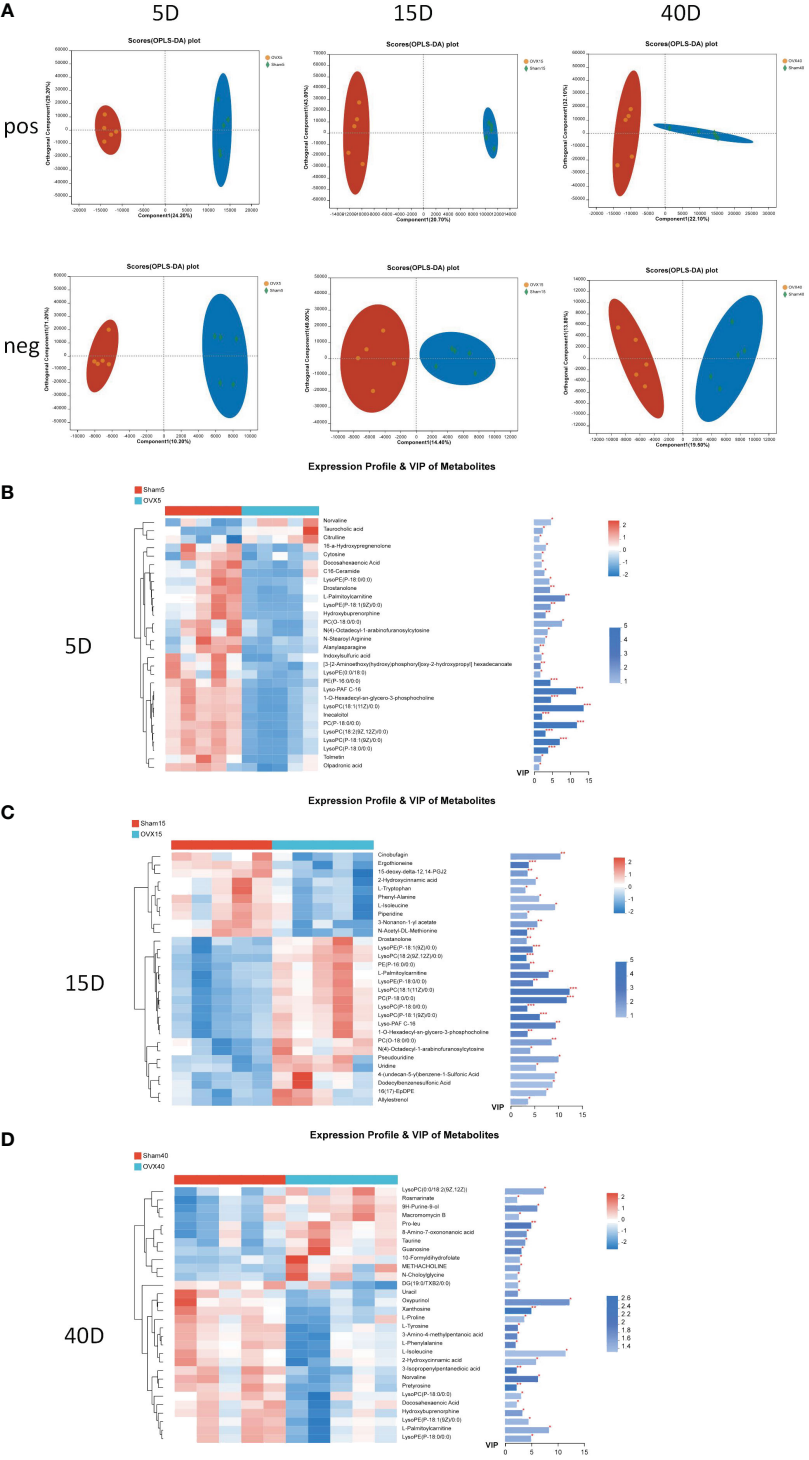
Multivariate statistical analysis of UPLC-MS data. **(A)** OPLS-DA score plot in positive and negative ion model 5, 15, and 40 days after surgery. Heatmap of the expression profile and VIP values of the top 30 metabolites according to the VIP values 5 days **(B)**, 15 days **(C)**, and 40 days **(D)** after surgery. *p < 0.05, **p < 0.01, and ***p < 0.001.

Multivariate statistical analysis method OPLS-DA was applied to select differential metabolites. The OPLS-DA score plots in positive and negative ion models 5, 15, and 40 days after surgery are shown in **Figure 2A**. The clustering trend indicated an obvious separation between the OVX group and Sham group 5, 15, and 40 days after surgery. Significant metabolites were selected by the condition that the VIP value was greater than or equal to 1 and the p value was less than 0.05. The heatmaps showed the expression of 30 significant metabolites between the OVX group and Sham group 5, 15, and 40 days after surgery, respectively (**Figures 2B–D**), which indicated the evident difference of metabolites between the OVX group and Sham group at this three time points. The VIP and p values of metabolites were presented on the side.

### 3.3 Metabolic pathways analysis and significant metabolite identification

**Figure 3:**
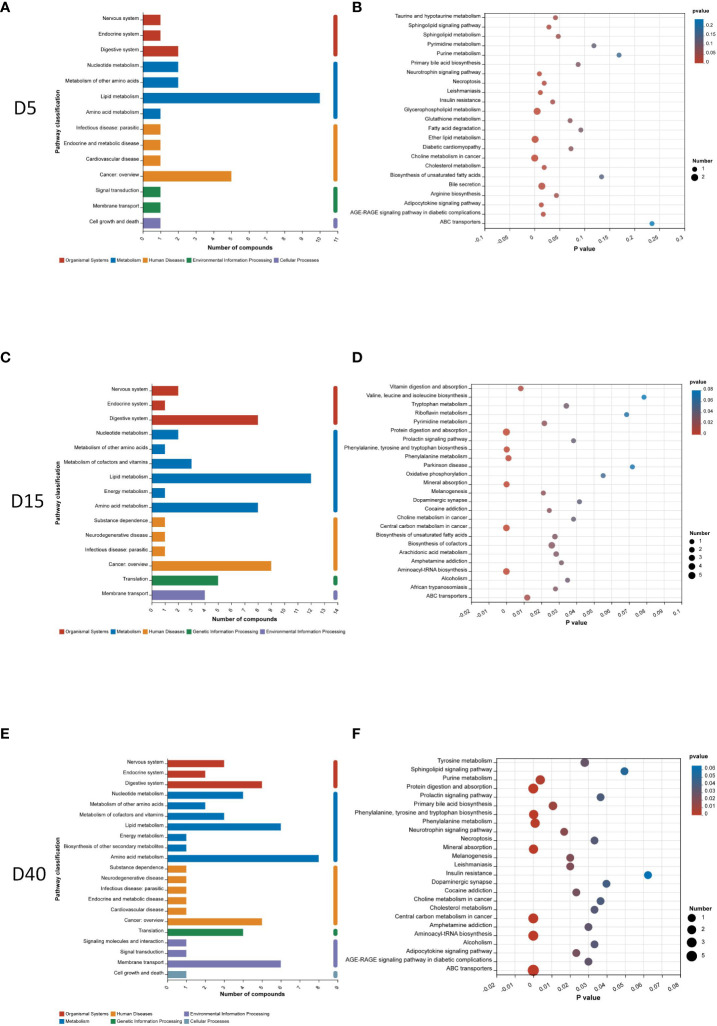
Pathway analysis of differential metabolites. Classification of metabolic pathways differential metabolites involving in 5 days **(A)**, 15 days **(C)**, and 40 days **(E)** after surgery. KEGG pathway enrichment analysis of differential metabolites 5 days **(B)**, 15 days **(D)**, and 40 days **(F)** after surgery.

In order to further investigate the potential pathway related with different stages of osteoporosis, metabolic pathway analysis was conducted. The classification of metabolic pathways, which differential metabolites at different time points involve in, illustrated that lipid metabolism was the most widely classified class of significant metabolites on the 5th and 15th days after surgery (**Figures 3A, C**). Amino acid metabolism was the most widely classified class of significant metabolites on the 40th day after surgery (**Figure 3E**). KEGG pathway enrichment analysis of differential metabolites on the 5th day showed 15 KEGG pathways with a p value less than 0.05, including sphingolipid signaling pathway, sphingolipid metabolism, insulin resistance, glycerophospholipid metabolism, ether lipid metabolism, cholesterol metabolism, arginine biosynthesis, adipocytokine signaling pathway, and bile secretion (**Figure 3B**). There were 21 KEGG pathways with a p value less than 0.05 identified on the 15th day. There are vitamin digestion and absorption; tryptophan metabolism; pyrimidine metabolism; protein digestion and absorption; phenylalanine, tyrosine, and tryptophan biosynthesis; phenylalanine metabolism; mineral absorption; biosynthesis of unsaturated fatty acids; arachidonic acid metabolism; and so on (**Figure 3D**). On the 40th day, 24 KEGG pathways with a p value less than 0.05 were identified, including tyrosine metabolism; sphingolipid signaling pathway; purine metabolism; protein digestion and absorption; phenylalanine, tyrosine, and tryptophan biosynthesis; phenylalanine metabolism; mineral absorption; cholesterol metabolism; adipocytokine signaling pathway; primary bile acid biosynthesis; and so on, which are shown in **Figure 3F**.

**Figure 4:**
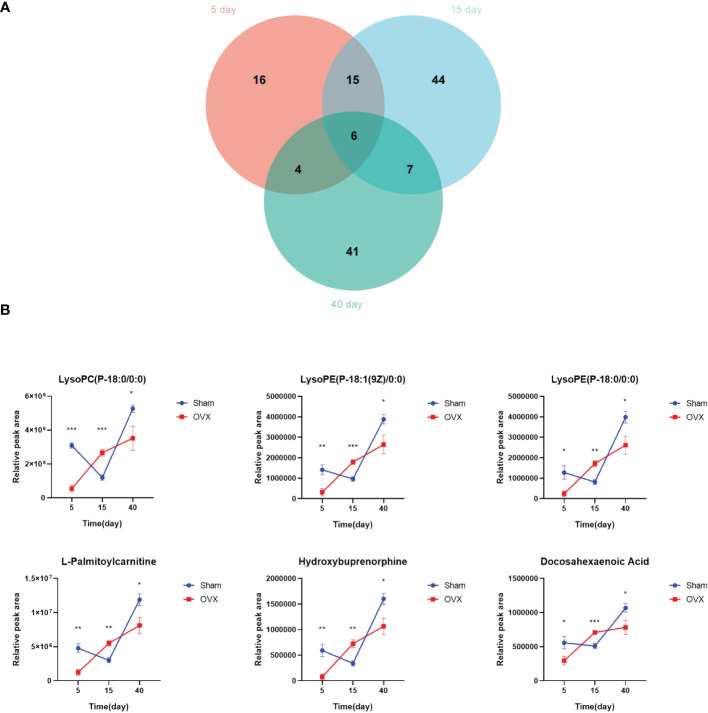
Screening for common differential metabolites. **(A)** Overlap between differential metabolites 5, 15, and 40 days after surgery. **(B)** Quantitative changes of six common differential metabolites. *p < 0.05, **p < 0.01, and ***p < 0.001.

The Venn diagram showed six common differential metabolites on the 5th, 15th, and 40th days after surgery (**Figure 4A**). They are lysoPC(P-18:0/0:0), lysoPE(P-18:1(9Z)/0:0), lysoPE(P-18:0/0:0), L-palmitoylcarnitine, hydroxybuprenorphine, and docosahexaenoic acid (DHA). Their quantitative changes are presented in **Figure 4B**. In the OVX group, the contents of these six common metabolites were significantly decreased on the 5th and 40th days after surgery compared with the Sham group (p < 0.05), whereas their content in the OVX group was significantly increased on the 15th day compared with the Sham group (p < 0.01).

### 3.4 Bone marrow lymphocyte subset analysis

**Figure 5:**
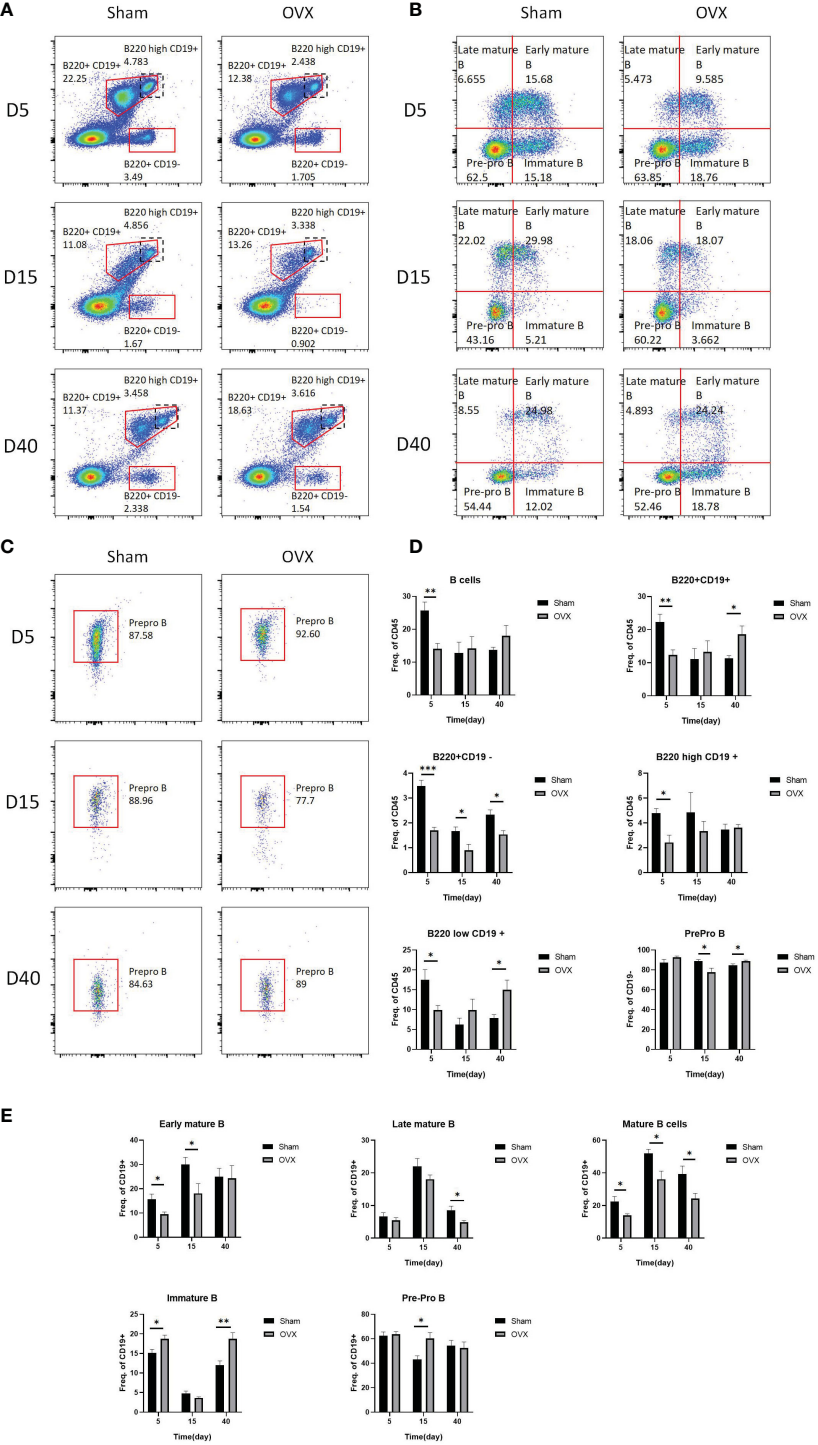
Representative FACS dots and proportions of B-cell subsets in the OVX group and Sham group at different time points. **(A)** Representative FACS dots of B220+CD19+ B cells, B220high CD19+ B cells, and B220+ CD19− B cells in the OVX group and Sham group at different time points. **(B)** Representative FACS dots of pre-pro B cells, early mature B cells, late mature B cells, and immature B cells in the OVX group and Sham group at different time points. **(C)** Representative FACS dots of prepro B cells in the OVX group and Sham group at different time points. **(D, E)** Proportions of B cells subsets in the OVX group and Sham group at different time points. *p < 0.05, **p < 0.01, and ***p < 0.001.

Bone marrow lymphocyte subsets also changed from the early stage of osteoporosis. The flow cytometric gating strategy of B cells and T cells is shown in **Supplementary Figure S1**. **Figures 5A-C** shows the representative fluorescence activated cell sorter (FACS) dots of B-cell subsets at different time points. The proportion of B cells, B220+CD19+ cells, B220+ CD19− cells, B220high CD19+ cells, and B220low CD19+ cells in the OVX group were significantly decreased on the 5th day after surgery compared with the Sham group. The proportion of B220+C19− cells in the OVX group was still significantly lower in the OVX group on the 15th and 40th days, but the proportion of B220+ CD19+ cells and B220low C19+ cells in the OVX group was significantly increased on the 40th day. The proportion of Prepro B cells was significantly decreased on the 15th day but increased on the 40th day in the OVX group after surgery (p < 0.05, **Figure 5D**). The proportion of early mature B cells was significantly decreased in the OVX group on the 5th and 15th days, and the proportion of late mature B cells was significantly decreased on the 40th day. The total proportion of early and late mature B cells in the OVX group was significantly decreased on these three time points compared with the Sham group, whereas the proportion of immature B cells in the OVX group was significantly increased on the 5th and 40th days after surgery (p < 0.05, **Figure 5E**). The proportion of pre-pro B cells in the OVX group was only significantly increased on the 15th day after surgery (p < 0.05, **Figure 5E**). We also investigated the subset of T cells and the representative FACS dots of CD4 cells and CD8 cells are shown in **Supplementary Figure S2A**. Compared with the Sham group, the proportion of CD4 T cells was significantly increased on the 15th day whereas the proportion of CD8 T cells was significantly decreased on the 5th and 15th days in the OVX group (p < 0.05, **Supplementary Figure S2B**).

### 3.5 Correlation analysis among common differential metabolites, lymphocyte subsets and bone microarchitecture

**Figure 6:**
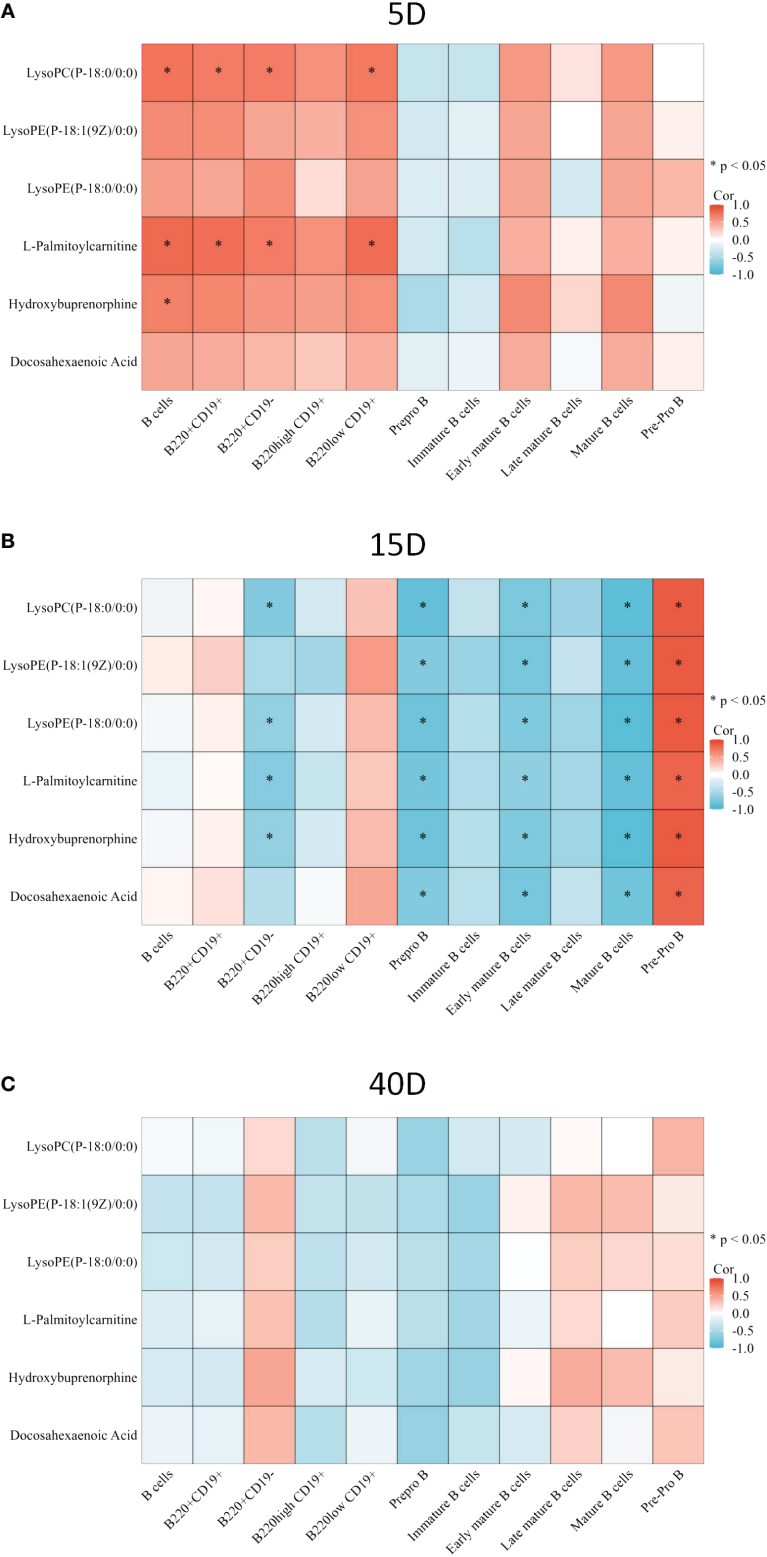
Correlation between common differential metabolite and B-cell subset proportion. **(A)** Correlation between common differential metabolite and B-cell proportion 5 days after surgery. **(B)** Correlation between common differential metabolite and B-cell proportions 15 days after surgery. **(C)** Correlation between common differential metabolite and B-cell proportions 40 days after surgery. The color of the square represents the value of the R. Red square means positive correlation, whereas blue square means negative correlation. *p < 0.05.

In order to explore the relationship between metabolites and lymphocyte subsets and the potential mechanism of bone loss, we applied correlation analysis. LysoPC(P-18:0/0:0) and L-palmitoylcarnitine were both positively correlated with the proportion of B cells, CD220+CD19+ cells, CD220+CD19- cells, and B220low CD19+ cells, and hydroxybuprenorphine was positively correlated with B cells on the 5th day after surgery (p<0.05, **Figure 6A**). On the 15th day after surgery, all the common differential metabolites were negatively correlated with the proportion of prepro B cells, early mature B cells, and mature B cells, whereas they were positively correlated with pre-pro B cells (p<0.05, **Figure 6B**). LysoPC(P-18:0/0:0), lysoPE(P-18:0/0:0), L-palmitoylcarnitine, and hydroxybuprenorphine were negatively correlated with the proportion of B220+CD19- cells (p<0.05, **Figure 6B**). There was no significant correlation on the 40th day after surgery (p>0.05, **Figure 6C**). In terms of T lymphocyte subsets, lysoPC(P-18:0/0:0) and L-palmitoylcarnitine were both positively correlated with CD8 on the 5th day (**Supplementary Figure S3A**, p<0.05). All the common differential metabolites were positively correlated with CD4 T cells (p<0.05) but negatively correlated with CD8 T cells on the 15th day (p<0.05, **Supplementary Figure S3B**). There was no significant correlation on the 40th day after surgery (p>0.05, **Supplementary Figure S3C**).

We also investigated the relationship between differential metabolites, lymphocyte subsets, and bone microarchitecture. There was no significant correlation between bone microarchitecture parameters and lymphocyte subset as well as common differential metabolites on the 5th day (p>0.05, **Supplementary Figure S4A, D**). On the 15th day after surgery, the proportion of B220+CD19− cells was positively correlated with Tb.N. The proportions of prepro B cells, late mature B cells, and CD8 T cells were negatively correlated with Tb.Sp. The proportion of late mature B cells was positively correlated BMD and Tb.Th. In addition, the proportions of mature B cells and CD8 T cells were positively correlated with Tb.Th, whereas the proportion of prepro B cells was negatively correlated with Tb.Th (p<0.05, **Supplementary Figure S4B**). All the common differential metabolites were both negatively correlated with Tb.Th, whereas lysoPE(P-18:0/0:0), L-palmitoylcarnitine and hydroxybuprenorphine were positively correlated with Tb.Sp (p<0.05, **Supplementary Figure S4E**). On the 40th day, prepro B cells were negatively correlated with BMD, BV/TV, and Tb.N. CD8 T cells were positively correlated with BV/TV and Tb.Th and negatively correlated with Tb.Sp (p<0.05, **Supplementary Figure S4C**). LysoPE(P-18:1(9Z)/0:0), hydroxybuprenorphine, and DHA were positively correlated with BMD, and all the common differential metabolites were positively correlated with Tb.N (p<0.05, **Supplementary Figure S4F**).

## 4 Discussion

Metabolomics is significant to the understanding of disease mechanism and development of novel diagnostic and therapeutic approaches. Osteoimmunology is also an evolving research direction in bone disease. In this study, we investigated the changes in bone metabolites and bone marrow lymphocytes, as well as their relationship, during the development of osteoporosis after the estrogen withdrawal. Significant changes in bone metabolites and bone marrow lymphocyte subsets were discovered, and there were also some significant correlations between common significant bone metabolites and lymphocyte subsets.

Metabolites are metabolic substrates and products driving basic cellular functions, which could provide specific characteristics associated with diseases (28). Bone metabolites have significantly changed since 5 days after ovariectomized surgery compared with the Sham group, which suggests that changes in bone metabolites happened before significant bone loss after estrogen deficiency. Lipid metabolism was the most widely classified class of significant metabolites on the 5th and 15th days after surgery, which suggests that lipid metabolism is critical in the early stage of the osteoporosis. Lipid metabolism reprogramming is found to be significant to the differentiation and activity of osteoclasts (29). Lipids and their derivatives are also a significant energy source of osteoblasts (30). Changes in lipid metabolism may be one of the important initiating factors in the development of osteoporosis. In addition, animal and human studies have also reported the importance of amino acid metabolism in bone, and many amino acids have been found to be related to bone density (31, 32). For example, studies have found that in postmenopausal women with decreased bone density, the content of glutamine increases significantly whereas the content of proline decreases (33, 34).

According to the results of KEGG pathway enrichments, many pathways associated with lipid were also found. The increasing number of enriched KEGG pathways with time indicates that the metabolic changes are increasing gradually as well. Cholesterol metabolism, adipocytokine signaling pathway, and sphingolipid signaling pathway were enriched on the 5th and 40th days after surgery. Insulin resistance and sphingolipid metabolism were enriched on the 5th day. The effect of cholesterol metabolism on bone metabolism is complex and depends on the forms of cholesterol according to a previous study (35). Sphingolipid and adipocytokines (including leptin and adiponectin) were both found to be one of the regulatory molecules in bone metabolism (36–38). In addition, there is a recent study indicating that there is a biphasic longitudinal relationship between bone mineral density and insulin resistance (39). Arachidonic acid metabolism and biosynthesis of unsaturated fatty acids were enriched on the 15th day after surgery. Arachidonic acids, a kind of omega-6 polyunsaturated fatty acids (PUFAs), are found to decrease the mineral apposition rate and promote osteoclastogenesis by the OPG/RANKL pathway. On the other hand, omega-3 PUFAs could preserve the bone loss caused by OVX surgery (40, 41).

There are also some other metabolic pathways enriched. Mineral absorption was significantly enriched on the 15th and 40th days after surgery, indicating that the damage of bone structure became obvious at the late stage after OVX surgery. Vitamin and protein digestion and absorption were enriched on the 15th day, and protein digestion and absorption were enriched on the 40th day, which indicates the importance of nutrition on the osteoporosis. A balanced diet and adequate intake of protein, calcium, and vitamin D play an important role in the prevention of osteoporotic fractures. Total protein intake was significantly positively correlated with bone microstructure, including bone density, bone strength, trabecula, and cortex (42, 43). On the 5th and 40th days after surgery, bili-related signaling pathways were enriched, including bile secretion and primary bile acid biosynthesis. Previous studies have found that bile acid synthesis is also a metabolic pathway related to bone density, because bile acid can affect intestinal calcium absorption (33).

Untargeted metabolomics have helped to discover many key metabolites and their relationship with the diseases (44). In our study, six metabolites, namely, lysoPC(P-18:0/0:0), lysoPE(P-18:1(9Z)/0:0), lysoPE(P-18:0/0:0), L-palmitoylcarnitine, hydroxybuprenorphine, and DHA, were significantly changed between the OVX and Sham groups 5, 15, and 40 days after surgery. Previous studies suggested that DHA, a kind of omega-3 PUFAs, could inhibit bone loss by inhibiting the osteoclastogenesis (45). The relationship between other common metabolites and bone metabolism needs further studies. Compared with the Sham group, the content of these six metabolites decreased significantly on the 5th and 40th days after surgery in the OVX surgery but increased significantly on the 15th day. It may be caused by metabolites being in a negative feedback state in order to maintain metabolic equilibrium on the 15th day after surgery.

Bone marrow lymphocyte subsets have also changed since 5 days after surgery. The proportion of mature B cells (including early mature B cells and late mature B cells) was decreased in the OVX group compared with the Sham group on the 5th, 15th, and 40th days after surgery, and the proportion of B cells was decreased on the 5th day. Previous studies suggested that B cells produced 64% of total bone marrow osteoprotegerin (OPG), which is important to the bone formation, and 45% of these OPG were derived from mature B cells (17). Decreased proportions of total B cells and mature B cells could result in decreased OPG and bone mass in turn. In the OVX group, the proportion of immature B cells was increased on the 5th and 40th days after surgery, and the proportion of pre-pro B cells was increased on the 15th day after surgery in the OVX group compared with the Sham group. Estrogen deficiency was found to increase the number of pre B cells and the concentrations of prostaglandin E2(PGE2). PGE2 could induce the expression of RANKL on the pre B cells, resulting in the increased osteoclastogenesis through RANKL–RANK interaction (46). In addition to OPG and RANKL, B cells could affect bone metabolism by other cytokines such as Wnt1, which is essential for bone formation. A previous study found that B220+ B cells could express Wnt1 and the proportion of B220+ B cells was decreased on the 5th day after estrogen deficiency in our study, which may be one of the factors initiating bone loss (47). T cells also play a significant role in the bone metabolism. The proportion of CD4 T cells was significantly increased on the 15th day whereas the proportion of CD8 T cells was significantly decreased on the 5th and 15th days in the OVX group. It has been reported that estrogen deficiency could dysregulate CD4 T cells and increase the secretion of TNF-α, RANKL, and so on to enhance the bone loss (48). On the other hand, CD8 cells were regarded to inhibit bone loss by secretion of OPG, IFN-γ, and so on (19).

Common significant metabolites may affect the subsets of the bone marrow lymphocytes to interfere with bone metabolism. On the 5th day after surgery, significant correlations between common significant metabolites and lymphocytes subsets began to appear. Metabolites could affect immune responses (49). It has been reported that metabolic pathways including lipid metabolism influence the development of B cells and T cells (50, 51). However, there was no significant bone microarchitecture changes, so there was no significant relationship between bone microarchitecture and metabolite and lymphocyte subsets. On the 15th day, bone microarchitecture had significantly changed, and there were significant correlations among bone microarchitecture and metabolite and lymphocyte subsets. On the 40th day, a significant relationship between metabolites and lymphocyte subsets disappeared, but there were still significant relationships between metabolite and lymphocyte subsets and bone microarchitecture. It suggests that metabolites may not interact with lymphocyte subsets at the late stage of osteoporosis, but metabolites and lymphocytes were still correlated with bone microstructure in other forms.

## 5 Conclusion

To conclude, significant changes have happened in bone metabolites and bone marrow lymphocyte subsets from the early stage of osteoporosis after estrogen deficiency before the significant bone loss. The changes were complicate. Lipid metabolism was significant in the initial process of bone loss. Metabolites may influence bone metabolism by interfering with lymphocyte subsets at the early stage of the osteoporosis. Understanding the effects of metabolites on immunity in bone microenvironment may offer new insights into the potential mechanism and provide novel therapies of osteoporosis.

## Data availability statement

The raw data supporting the conclusions of this article will be made available by the authors, without undue reservation.

## Ethics statement

The animal study was approved by The Animal Ethics Committee of Shanghai Tenth People’s Hospital. The study was conducted in accordance with the local legislation and institutional requirements.

## Author contributions

SW: Data curation, Formal analysis, Writing – original draft. YQ: Formal analysis, Writing – original draft, Funding acquisition. CT: Project administration, Writing – review & editing. HT: Project administration, Writing – review & editing. JL: Project administration, Writing – review & editing. JC: Project administration, Writing – review & editing. LZ: Conceptualization, Writing – review & editing. GT: Conceptualization, Writing – review & editing, Funding acquisition.
