# Supplementary material for: Early changes of bone metabolites and lymphocyte subsets may participate in osteoporosis onset: a preliminary study of a postmenopausal osteoporosis mouse model
Source: Front Endocrinol (Lausanne). 2024 Feb 28;15:1323647. doi: 10.3389/fendo.2024.1323647 (PMC10933021; doi:10.3389/fendo.2024.1323647)

Supplementary Table S1 List of antibodies used for flow cytometry analysis.

**Antibodies**	**Source**	**Clone**	**Cat.No**
R718 Rat Anti-Mouse CD45	BD	I3/2.3	567464
Brilliant Violet 605™ anti-mouse IgM Antibody	BioLegend	RMM-1	406523
Brilliant Violet 650™ anti-mouse CD19 Antibody	BioLegend	6D5	115541
PE Rat Anti-Mouse CD43	BD	S7	561857
PerCP-Cy™5.5 Rat Anti-Mouse CD45R/B220	BD	RA3-6B2	552771
APC/Cyanine7 anti-mouse IgD Antibody	BioLegend	11-26c.2a	405715
PE-CF594 Hamster Anti-Mouse CD3e	BD	145-2C11	562286
APC-Cy™7 Rat Anti-Mouse CD4	BD	GK1.5	552051
BV650 Hamster Anti-Mouse TCR β Chain	BD	H57-597	742483
PerCP-Cy5.5 Rat Anti-Mouse CD8b	BD	H35-17.2	567597

Supplementary Figure S1 The flow cytometric gating strategy of B cells (A) and T cells (B).

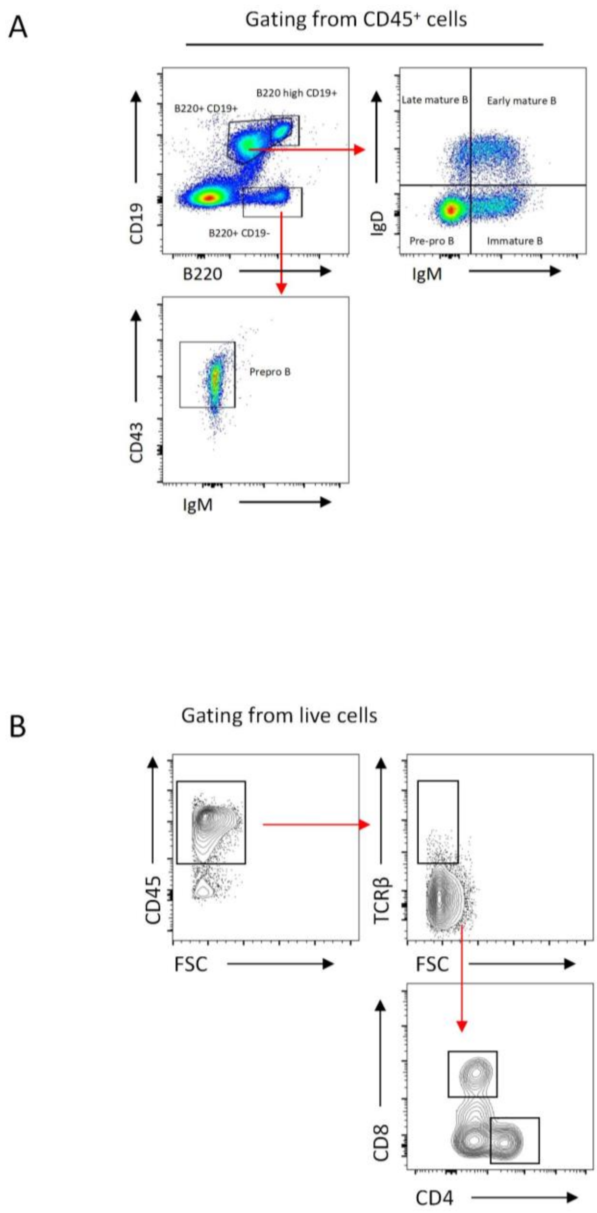

Supplementary Figure S2. Representative FACS dots of CD4 cells and CD8 cells 5, 15 and 40 days after surgery(A). Proportion of CD4 cells and CD8 cells 5, 15 and 40 days after surgery between OVX and Sham groups(B). * $p < 0.05$, ** $p < 0.01$, and *** $p < 0.001$.

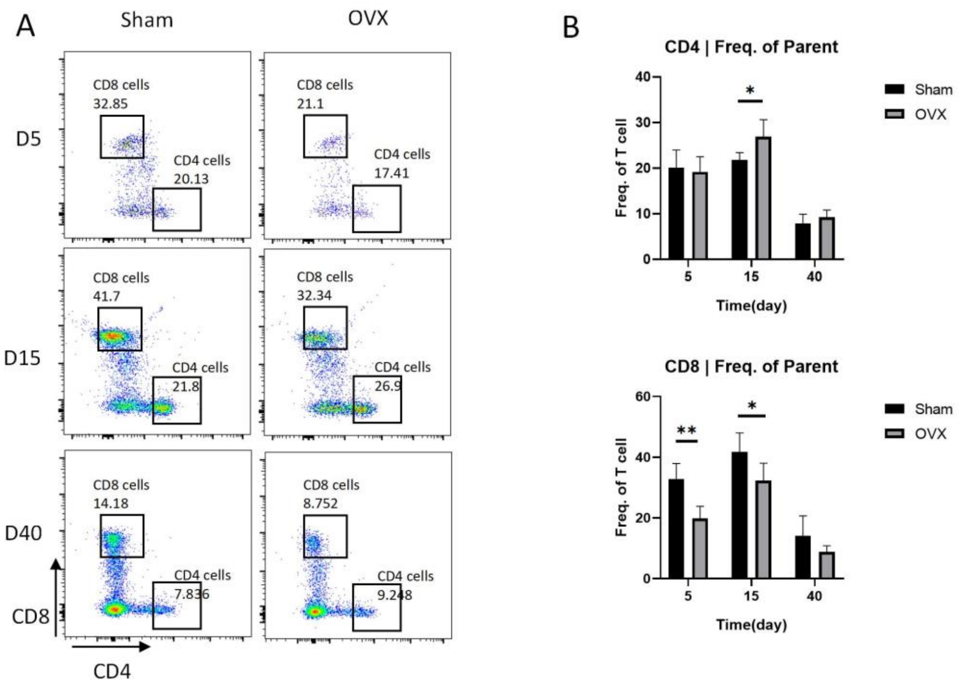

Supplementary Figure S3. Correlation between common differential metabolites and T cells subsets proportion 5 days(A), 15 days(B) and 40 days(C) after surgery. * $p < 0.05$, ** $p < 0.01$, and *** $p < 0.001$.

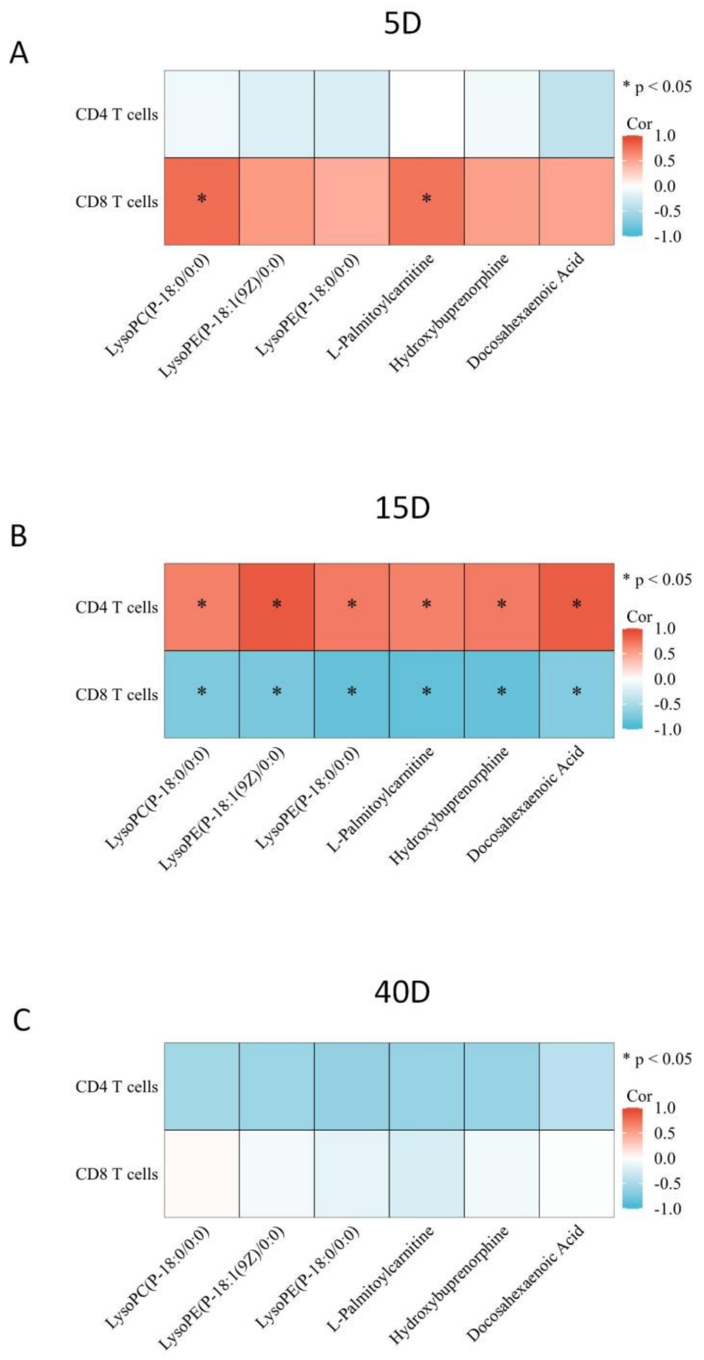

Supplementary Figure S4. Correlation between common differential metabolites and bone microarchitecture parameters 5 days(A), 15 days(B) and 40 days(C) after surgery. Correlation between T and B cells subsets proportion and bone microarchitecture parameters 5 days(D), 15 days(E) and 40 days(F) after surgery. * p < 0.05, ** p < 0.01, and *** p < 0.001.

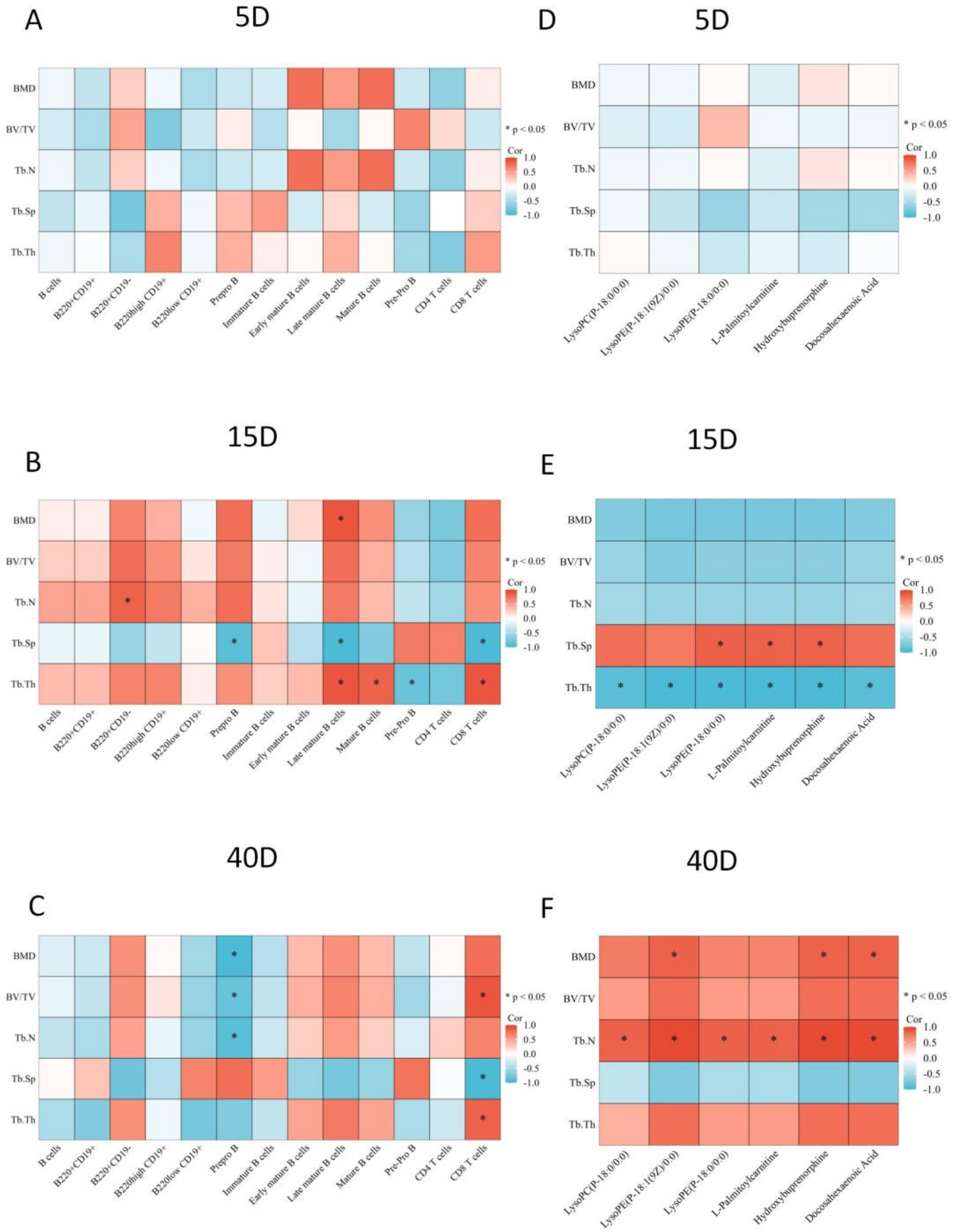

Supplement: Supplementary file 1 [file DataSheet_1.pdf]
